# Covid-19 in the Caribbean: lessons learned from the ongoing international medical and scientific cooperation

**DOI:** 10.1186/s12992-021-00706-3

**Published:** 2021-05-10

**Authors:** Dabor Resiere, Hossein Mehdaoui, Hedda Dyer, Cyrille Chabartier, André Cabié, Jocelyn Inamo, Keats Compton, Rémi Neviere, Bruno Megarbane, Hatem Kallel

**Affiliations:** 1 Intensive Care Unit, University Hospital of Martinique, CS 97261, Fort-de-France Cedex, 97200 Fort-de-France, Martinique; 2 Departments of Surgery, Ross University, Lloyd Erskine Sandiford Centre, Bridgetown, Barbados; 3 Department of Infectious Diseases, University Hospital of Martinique, CS 97261, Fort-de-France Cedex, Fort-de-France, Martinique; 4 Department of Cardiology, University Hospital of Martinique, CS 97261, Fort-de-France Cedex, Fort-de-France, Martinique; 5 Department of Medical and Toxicological Critical Care, Lariboisière Hospital, Paris-Diderot University, 2 rue Ambroise Paré, 75010 Paris, France; 6 Intensive Care Unit, Cayenne General Hospital, Av des Flamboyants, 97306 Cayenne, French Guiana

**Keywords:** Covid-19, Coronavirus, International medical cooperation, Regional healthcare systems, Caribbean

## Abstract

The coronavirus disease (Covid-19) crisis presents as human, social and economic challenges. The advent of Covid-19, unfortunate as it is, has highlighted the need for close medical cooperation between states. Medical cooperation is the key counter to fight against the Covid-19 pandemic.

## Background

In recent years, there has been a growing awareness of emergent outbreaks of tropical Diseases in the Caribbean. It was hoped that the lessons learned during these epidemics would have served as an impetus for a revised approach in the strategic planning for the management of infectious diseases in the Caribbean.

As the coronavirus spreads rapidly across the Caribbean, at-risk countries had to prepare for adequate hospital capacity. With competing priorities and smaller populations relative to most developed countries, it has been challenging to plan for Intensive Care Units (ICUs) to cater to pandemics. Indeed, increased ICU capacity requires increased infrastructural and human resources, i.e. fully trained staff and equipment. Furthermore, Covid-19 has impacted all social strata, affecting industry, manufacturing, tourism, labor and education. From metropolitan cities to small villages, large numbers of people have had to isolate themselves.

The Caribbean region is not immune. With the increased prevalence of chronic non-communicable diseases and increased mortality rates in patients with underlying health issues, the importance of robust primary healthcare programs should be the driving force for a regional a systematic approach, catering to the unique needs of the Caribbean countries. Building capacity for increased scope of disaster management as well as training and teaching are important lessons from Covid-19.

In this commentary, we discuss the potential impact of international medical cooperation in the region, and how should we work together to fight against the pandemic.

## Main text

The first Covid-19 case in the Caribbean was confirmed at the beginning of March 2020 [1]. As the coronavirus spread rapidly across the Caribbean, at-risk countries needed to prepare for adequate hospital capacity. However, the virus has now spread all over the area, though with limited fatalities, probably due to the prompt closure of their frontiers (both land and sea), as well as varying degrees of lockdowns and curfews, realizing that denying entry to carriers would reduce community spread of the virus.

The longer-term deployment of health care measures for tackling the virus include additional ICU staff, acquisition of beds, equipment, ventilators, catheters, etc. … pose significant challenges for small countries faced with competing for budgetary constraints, unlike most developed countries. Increased ICU capacity requires increased infrastructural and human resources, e.g. fully trained staff, and equipment. It is thus a major challenge for ICUs to plan for pandemics. Consultation of expert clinical advisors and access to additional funding to achieve these goals are extremely important. The region continues to rely on receiving assistance and consultancy expertise from traditional historic alliances, which may not always be forthcoming.

The Covid-19 crisis presents not only as human, social and economic challenges globally [2–4], but demonstrates just how interdependent we all are. Prior to the advent of Covid-19, the world had been visited by smaller scale epidemics, prompting some countries to enact management plans, which they retained for future use as required. These countries had a head start in their response, once Covid-19 went global. That event, coupled with the increasing incidence of chronic non-communicable diseases (NCDs), the increased mortality in patients with underlying health issues, the increasing frequency and severity of natural disasters, as well as the recent emergence of tropical diseases, should have alerted the Caribbean region to the fragility of their healthcare systems, and highlighted the need for robust primary healthcare programs. It was hoped that the lessons learned during these epidemics would have served as an impetus for a revised approach in the strategic planning for the management of infectious disease in the Caribbean.

The sheer scale of Covid-19 will hopefully emphasize the need, on a regional basis, for: capacity building; data gathering and analysis; placing a higher priority on healthcare in budgetary allocation (largely absent) – as at August 14, 2020, the Caribbean region has already registered more than 131,089 confirmed cases among 45.29 million people living across the region and a growing, though still small number of fatalities [5].

During the Covid-19 crisis, the measures taken to contain and slow the spread of the virus, such as border closures and lockdowns, have had severe adverse effects on the global economy. Individual governments have had to strike a balance between implementing these measures and preventing the collapse of their economies, in the face of healthcare systems and social safety nets stretched beyond capacity. In the Caribbean, fragile healthcare systems could face collapse. The tourism sector (primary economic engine) is at a standstill, social safety nets are virtually non-existent, especially for people in the informal economy, thus governments will be under pressure to give higher priority to relaunching economic activity than to a collective, strategic planning approach to the provision of healthcare on a regional basis. An impact scenario showed that the slump in tourism caused a fall in total gross domestic product growth by 8% in the Caribbean [6].

The current pandemic is nowhere near being over, and thus provides an opportunity for the region to define a Caribbean approach to this and future such events. The immediate priority should be to secure financing for the acquisition of equipment for initial responses: personal protective equipment for frontline staff, testing kits, diagnostic equipment and drug treatments, and importantly, tracing and tracking software. The logistics of distribution need to be defined for both air and sea delivery. The Organization of Eastern Caribbean States (OECS) already maintains a successful Pharmaceutical Procurement Program, which could no doubt be expanded to include member states of the wider Caribbean community (CARICOM).

Covid-19 has demonstrated the value of medical cooperation on a worldwide basis. In this context, the Caribbean Medical Doctors association (CDA) played a major role in sharing the epidemiological spread of Covid-19 in the region, experience exchange, and capacity-building in individual territories. CDA was created in 2000 in Miami, Florida by a group of doctors from various countries. It’s a non-governmental, independent, non-profit organization registered at the “Journal officiel de la République Française” (N°1895/20020024). Its objective is to share clinical experiences not just within the region, but with colleagues in the United States of America, Europe, and Africa. The group has so far attracted 250 medical professionals using social media to host webinars. The group believes that increased use of technology needs to be explored to facilitate closer medical co-operation for healthcare delivery as we enter into a new world of pandemics and closed frontiers. Technology will also play a major role in the capacity building (training, teaching, human resource development) referred to above [7].

The Covid-19 pandemic has presented yet another opportunity for Caribbean healthcare policymakers to engage in multilateral cooperation, so as to build robust health care systems. CARICOM, the Pan American Health Organization (PAHO), the Caribbean Public Health Agency (CARPHA) and the Organization of Eastern Caribbean States (OECS) worked together to contain and manage the epidemic. This allowed equipment transfers by friendly governments for expansion of ICU capacity (e.g. ventilators and beds). In addition, OECS and CDA helped in the coordination of routine medical referrals or emergency medevac from neighbouring islands to Martinique and Guadeloupe. Indeed, prior to the pandemic, regional organisations may have focused mainly on policy positions. The pandemic led to a practical involvement in capacity-building and patients medevac to referral centers.

Such cooperation needs to be maintained as a permanent feature of regional healthcare provision. Both regional and global co-operation among governments and international agencies will be key to mitigating the virus spread [8]. A coordinated response to Covid-19 is the most efficient way to fight the virus. A notable example of medical co-operation is that of German and Swiss hospitals that receive patients from various European countries [8].

The Caribbean region, being heavily dependent on tourism, has suffered economically, but has fared better on the health impact of this pandemic. With the exception of the Dominican Republic, which receives the largest number of foreign visitors, the islands were able to forestall the importation of the virus by the early closure of their frontiers, thus minimizing community spread. The results present a stark difference – DR, 39,588 confirmed, 20,056 recovered, 829 deaths (Jul 09); islands, less than 20 deaths.

Among two-hundred and twenty patients admitted to the University Hospital of Martinique (February 1st, 2020 - June 1st, 2020), only 42 patients with a positive SAR-CoV-2 real-time reverse transcriptase-PCR (RT-PCR), were hospitalized in ICU for severe critical illness. Nevertheless, 7 out of 42 admitted patients were transferred from another Caribbean hospital. Long-term strategic planning to build both infrastructural and human resource health care capacity is essential.

The Caribbean region must ensure that the development of their native human resource and accompanying healthcare infrastructure is prioritized to guarantee self-reliance on an ongoing basis, as well as during pandemics. The Covid-19 was an illustration of solidarity in enhancement and building of healthcare capacity thanks to cooperation between governments, the CARICOM, the PAHO, the CARPHA, the OECS, and the CDA. This interface between disease, politics, and non-government organizations was the key to face the Covid-19 pandemic and the way to strengthen the healthcare system in the Caribbean.

## Conclusion

The Covid-19 pandemic has demonstrated, unequivocally, the value of multilateralism. It has revealed an urgent need to expand capacity for human resources and healthcare infrastructure at the national level. This two-pronged approach may be the only effective method to ensure the sustainability of improved healthcare, and to save lives during pandemics, particularly in the Caribbean region.

## Data Availability

Not applicable.
